# The Effect of Natural Multidecadal Ocean Temperature Oscillations on Contiguous U.S. Regional Temperatures

**DOI:** 10.1371/journal.pone.0131349

**Published:** 2015-06-22

**Authors:** Bruce E. Kurtz

**Affiliations:** Independent consultant, Bradenton, Florida, United States of America; University of Vigo, SPAIN

## Abstract

Atmospheric temperature time series for the nine climate regions of the contiguous U.S. are accurately reproduced by the superposition of oscillatory modes, representing the Atlantic multidecadal oscillation (AMO) and the Pacific decadal oscillation (PDO), on a monotonic mode representing, at least in part, the effect of radiant forcing due to increasing atmospheric CO_2_. The relative importance of the different modes varies among the nine climate regions, grouping them into three mega-regions: *Southeastern* comprising the South, Southeast and Ohio Valley; *Central* comprising the Southwest, Upper Midwest, and Northeast; and *Northwestern* comprising the West, Northwest, and Northern Rockies & Plains. The defining characteristics of the mega-regions are: *Southeastern* – dominated by the AMO, no PDO influence; *Central* – influenced by the AMO, no PDO influence, *Northwestern* – influenced by both the AMO and PDO. Temperature vs. time curves calculated by combining the separate monotonic and oscillatory modes agree well with the measured temperature time series, indicating that the 1938-1974 small decrease in contiguous U.S. temperature was caused by the superposition of the *downward*-trending oscillatory mode on the *upward*-trending monotonic mode while the 1980-2000 large increase in temperature was caused by the superposition of the *upward*-trending oscillatory mode on the *upward*-trending monotonic mode. The oscillatory mode, mostly representing the AMO, was responsible for about 72% of the entire contiguous U.S. temperature increase over that time span with the contribution varying from 86 to 42% for individual climate regions.

## Introduction

The atmospheric temperature of the contiguous U.S. has increased by about 1.0 K since 1910, but not at a uniform rate, increasing between 1910 and 1938, decreasing slightly between 1938 and 1974, increasing between 1974 and 2000, and decreasing slightly since 2000. The rate of temperature increase between 1980 and 2000 was 0.029 K yr^-1^ and since 2000 has been near zero. A similar pause in the rate of global warming has been reported and has attracted a great deal of attention with many explanations proposed. These are summarized in a recent article by Chen & Tung [1] and are mostly focused on change in radiative forcing due to volcanic or anthropogenic aerosols or change in ocean heat content. The latter is a reasonable explanation since, compared to the atmosphere, the ocean is able to store very large amounts of heat with little change in temperature (the heat capacity of the entire atmosphere is equal to that of only a few meters of ocean), but no fully satisfactory explanation has been offered for the mechanism by which heat might be captured or why it should be captured now but was not between 1980 and 2000.

It may be that the current pause in contiguous U.S. warming and the pause seen between 1938 and 1974 have the same root cause, a regular change in the North Atlantic sea surface temperature (SST) with a period of 60–80 years known as the Atlantic multidecadal oscillation (AMO). In 2000, just as in 1938, the AMO may have ended a decades-long warming stage that had been contributing to the increase in atmospheric temperature and begun a new cooling stage, causing the stalling of atmospheric temperature increase. The AMO was not widely recognized prior to an article in 2000 by Kerr [2], which suggested that the rapid global warming seen during 1980–2000 was caused by superposition of a natural mode of temperature variation due to the AMO and an anthropogenic mode due to increasing atmospheric CO_2_. Subsequent research has increased support for this idea [3–6] and it is now accepted that the AMO has important effects on regional and global climate [7–10].

Schlesinger and Ramankutty [11] identified the AMO in 1994 using spectrum analysis of temperature records from 1858–1992. The subsequent paper by Kerr coined the term "AMO" and referenced work by Delworth and Mann in 2000 [12] that used proxy-based reconstructions of surface temperatures from the past 330 years to identify a temperature oscillation with a period of approximately 70 years and suggested variation in the Atlantic meridional overturning circulation (AMOC) as the cause. In 2001 Enfield et al. [13] reported a 65–80 year cycle in North Atlantic SST data for the years 1856–1999 and in 2004 Gray et al. [14] created a tree-ring based reconstruction of the AMO going back to 1567 with a 60–100 year cycle, tying this to SST anomalies from Kaplan et al. [15]. In 2005 Sutton and Hodson [16] showed evidence for the AMO using an 1871–2003 index based on annual mean SST observations and Polyakov et al. [17–18] reported a 50–80 year ocean surface temperature cycle that is exceptionally strong in the Arctic. In 2011 Knudsen et al. [19] used climate proxy data to suggest that a 55–70 year AMO has persisted for at least the last 8000 years.

In 2013 Macias et al. [20] analyzed recent warming of Mediterranean waters and concluded that more than half of the warming was attributable to a sinusoidal mode resembling the AMO superposed on a presumably anthropogenic monotonic component, with the recent slowdown in warming due to the end of AMO warming. In 2014 the same authors [21] used singular spectrum analysis to examine the global warming pause and identified three periods with very little warming: 1878–1907, 1945–1969, and 2001-onwards. These periods coincide with the superposition on the monotonic temperature trend of the warming stage of a multidecadal variation in global atmospheric temperatures resembling the AMO.

In 2015 Yao et al. [22] described the global warming pause as a “natural product of interactions of a secular warming trend and a multidecadal oscillation” resulting in warming slowdowns in the middle of the twentieth and the beginning of the twenty-first centuries. Using empirical mode decomposition applied to SST and atmospheric temperature time series for 1880–2012, they found that these slowdowns were caused by the superposition of the AMO and PDO on the steady warming presumably caused by increasing amounts of atmospheric CO_2_.

It is generally accepted that the AMO is caused by regular variation in the flow rate of the AMOC, which was described in 1959–61 by Stommel and Arons [23–24] as a global circulation comprising northward flow in the upper layer of the Atlantic Ocean ending in deepwater formation in extreme northern latitudes, balanced by southward abyssal return flow to upwelling in ocean basins contiguous with the Southern Ocean. The AMOC is the primary means for transporting heat from the Atlantic equatorial region to northern latitudes [25]. There is as yet no experimental verification that the AMO is caused by multidecadal variation in the AMOC flow because accurate flow measurements from the RAPID moored array at 26.5°N [26] have only been available for a short time. If the AMO is caused by a regular variation in AMOC flow that has persisted for thousands of years, the variation must be driven by a robust mechanism internal to the ocean [27–29] rather than by ocean-atmosphere interaction, as the latter would be subject to stochastic influences inconsistent with this long-term regularity. Not much has been offered that would explain an internal ocean mechanism behaving in this way, but it has recently been proposed that the AMOC flow oscillation could be caused by the interaction of AMOC thermal resistance and North Atlantic upper layer thermal capacitance by analogy to an electrical resistance-capacitance network [30].

**Fig 1:**
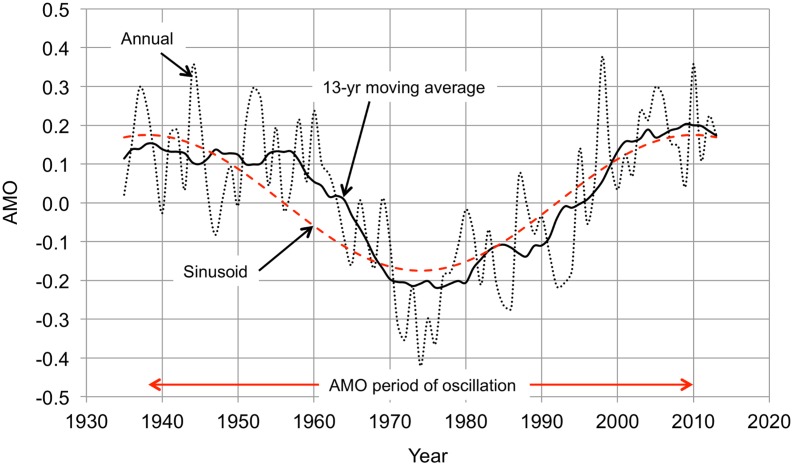
Atlantic multidecadal oscillation (AMO) 1935–2013. The AMO is the difference between the North Atlantic sea surface temperature (SST) and its trend line (slope equal to 0.0026 K yr^−1^ for 1856–2013). The annual measurements (black dotted line) are smoothed with a 13-year centered moving average filter (black solid line). The sinusoid (red dashed line) is a least squares fit to the moving average. The data are from the NOAA Earth System Research Laboratory (ESRL) AMO website, http://www.esrl.noaa.gov/psd/gcos_wgsp/Timeseries/AMO/.

**Fig 2:**
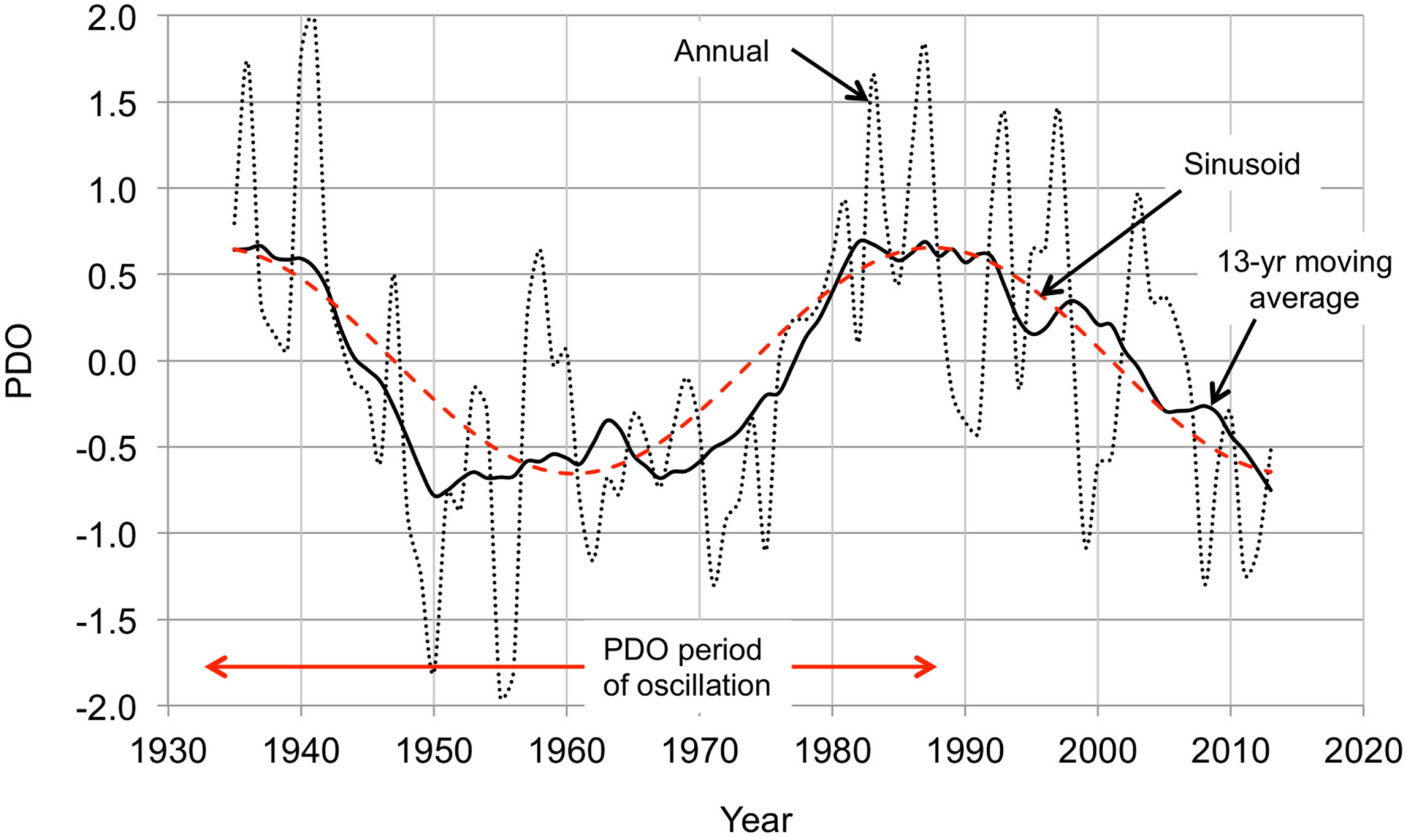
Pacific decadal oscillation (PDO) 1935–2013. The PDO is the leading principal component of the North Pacific monthly sea surface temperature. The annual measurements (black dotted line) are smoothed with a 13-year centered moving average filter (black solid line). The sinusoid (red dashed line) is a least squares fit to the moving average. The data are from the University of Washington's PDO website, http://jisao.washington.edu/pdo/.

Fig 1 shows the AMO for 1935–2013, which captures a full period of the oscillation. The sinusoid fitted to the data has a peak at 1938 and a period of 72 years. The AMO is usually described as being either in a cool stage when it is negative or in a warm stage when it is positive, but It is more useful to describe the AMO as being in one of three stages: cooling, warming, or neutral. If the AMO neutral stage is arbitrarily defined as SST within 80% of the peak value (positive or negative) of the sinusoid, then 1945–1966 was a cooling stage and 1981–2002 a warming stage. These classifications are inexact because the transitions between stages are gradual, but they are helpful in recognizing that the AMO warming stage adds to (and the cooling stage subtracts from) the steady warming presumably caused by increasing atmospheric CO_2_. Fig 2 shows another ocean temperature oscillation, much less prominent than the AMO but known to have important regional influences. This is the Pacific decadal oscillation (PDO). Major changes in northeast Pacific ecosystems have been linked to the PDO, but its cause is unknown, it is much less predictable than the AMO, and its effects are more localized [31]. The 1935–2013 time span captures nearly three PDO half-cycles. The sinusoid fitted to the data has a peak at 1933 and a period of 54 years.

**Fig 3:**
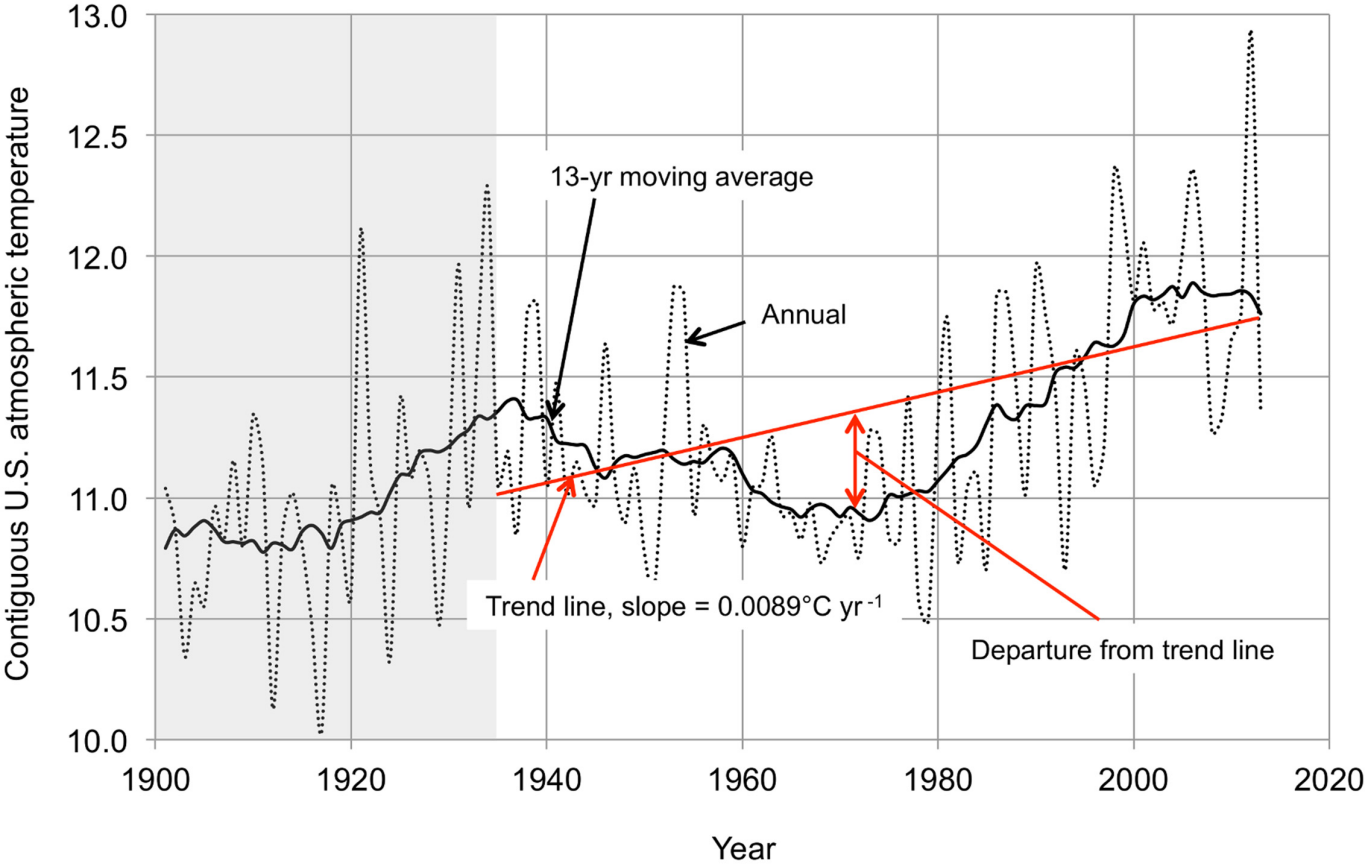
Contiguous U.S. atmospheric temperature history 1902–2013. The black dotted line is the annual atmospheric temperature time series (°C). The black solid line is the 13-year centered moving average. The 1935–2013 trend line is in red. The data are from the NCDC website, http://www.ncdc.noaa.gov/cag/time-series/us.

**Fig 4:**
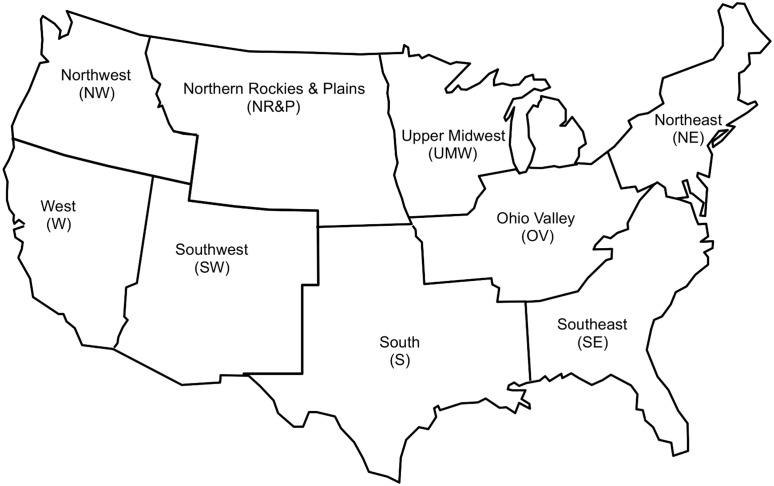
Contiguous U.S. NCDC climate regions. The figure shows the approximate locations of the climate regions and is intended only for illustrative purposes. An accurate map showing the exact locations of the climate regions is available at http://www.ncdc.noaa.gov/monitoring-references/maps/us-climate-regions.php.

Fig 3 shows the atmospheric temperature time series for the entire contiguous United States. The same kinds of temperature time series are available for the nine individual climate regions within the contiguous U.S. identified by the NOAA National Climatic Data Center (NCDC) as “climatically consistent.” Fig 4 shows the approximate locations of these climate regions. The four regions along the U.S. southern boundary—West (W), Southwest (SW), South (S), and Southeast (SE)–are aligned in a fairly regular longitudinal progression, as are the four regions along the northern border—Northwest (NW), Northern Rockies & Plains (NR&P), Upper Midwest (UMW), and Northeast (NE). The Ohio Valley (OV) region occupies a unique position, pushed southward by the Great Lakes so as to intrude on the S and SE regions.

## Methods

Superposed oscillatory and monotonic modes of temperature variation are used to represent the contiguous U.S. temperature time series, based on findings supporting this representation for global temperatures described in the preceding section. The oscillatory modes represent ocean temperature oscillations and the monotonic mode represents, at least in part, the effect of increasing atmospheric CO_2_. The method used is a variant of principal component analysis (PCA) adapted for use with non-linear data sets. Traditional PCA uses an orthogonal linear transformation that is not suitable for identifying physically meaningful relationships explaining the behavior of this highly non-linear 1935–2013 temperature time series. The shortness of the data set precludes the use of singular spectrum analysis. A suitable method is non-linear or generalized principal component analysis (GPCA), which identifies orthogonal coordinates that fit the data to algebraic curves (or surfaces) by minimizing the sum of the squares of the deviations from the curves [32]. The monotonic and oscillatory transformations were chosen because, based on their success in explaining global temperature change, they should be physically meaningful. See S1 File.

The starting point for the analysis of the time temperature series for the contiguous U.S. (in its entirety and for the individual climate regions) is the assumption that they can be described in terms of three modes (principal components): two oscillatory modes derived from the sinusoids fitted to the AMO and PDO temperature time series (Figs 1 and 2) and a monotonic (linear) mode with a constant slope derived from the atmospheric temperature time series trend line (Fig 3). The computation is straightforward: the 1935–2013 atmospheric temperature time series data are smoothed with a 13-year moving average, the trend line (monotonic mode) is subtracted from the measured data to give the departures from the monotonic mode, and the correlations of the departures with the monotonic and oscillatory modes (the sinusoids of Figs 1 and 2) are calculated. In most cases the monotonic and AMO oscillatory modes together account for almost all of the variation without taking into account the PDO. Where the influence of the PDO is significant the two oscillatory modes are combined to create a single oscillatory mode, adjusting the PDO sinusoid contribution to maximize the correlation between the departures from the monotonic mode and the oscillatory mode. The monotonic and oscillatory modes are then combined, adjusting the amplitude of the oscillatory mode to maximize the correlation between the temperature time series and the combined function.

## Results and Discussion

**Fig 5:**
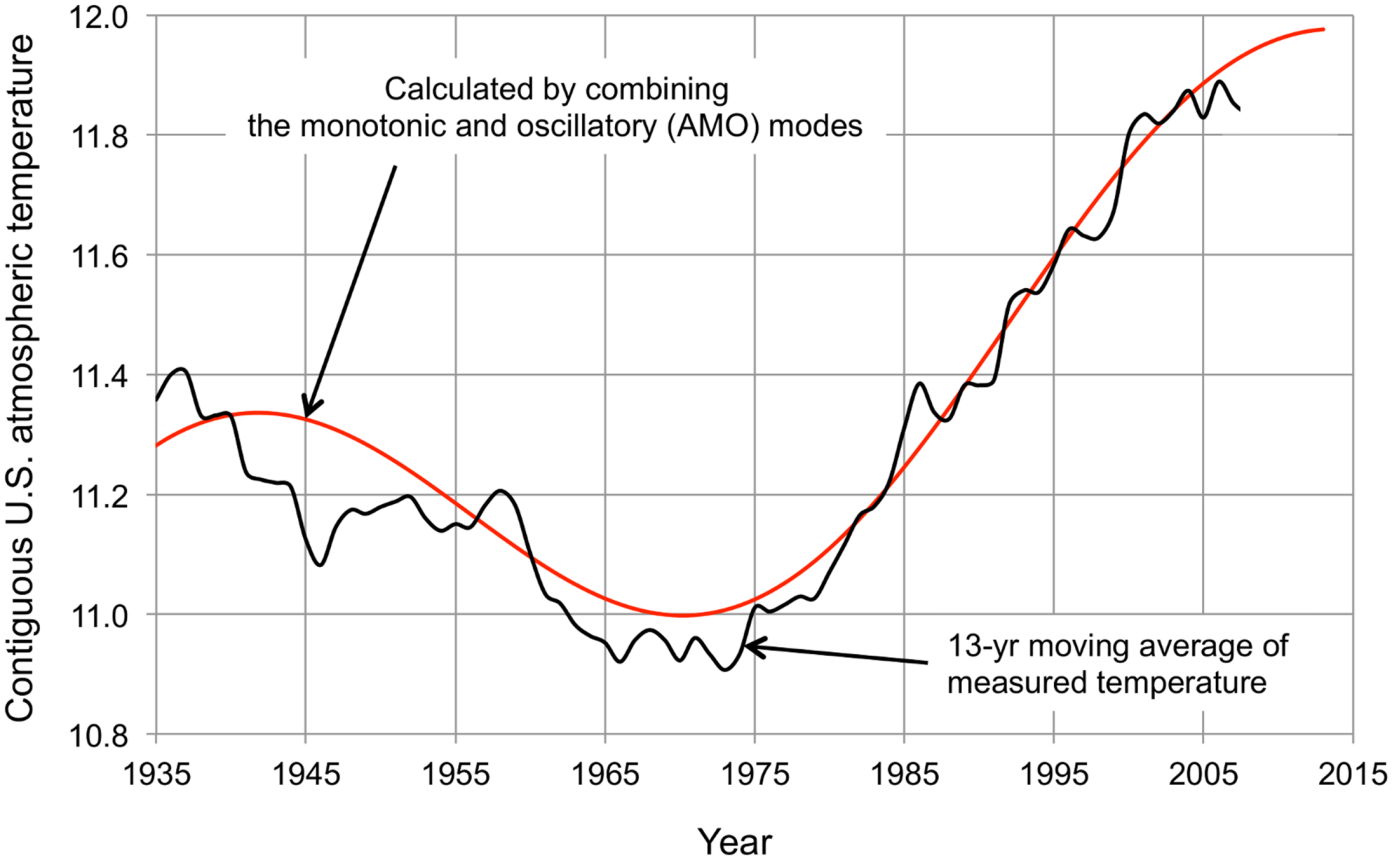
Measured and calculated contiguous U.S. temperature change. The black solid line is the 13-year centered moving average atmospheric temperature (°C) from Fig 3 (truncated at 2007 because of the 13-year moving average). The red solid line is calculated by combining the monotonic and oscillatory (AMO) modes. The slope of the monotonic mode is 0.0089 K yr^-1^ and the amplitude of the oscillatory mode (AMO) is 0.75 K. The correlation between the measured and calculated curves is 0.98.

Fig 5 shows the calculated curve of temperature vs. time for the entire contiguous U.S., obtained by combining the monotonic and oscillatory (AMO) modes. The correlation with the measured temperature time series is 0.98 and is not significantly improved by including the PDO oscillatory mode. Similar curves are calculated for each of the NCDC climate regions and are the basis for the results that follow.

**Fig 6:**
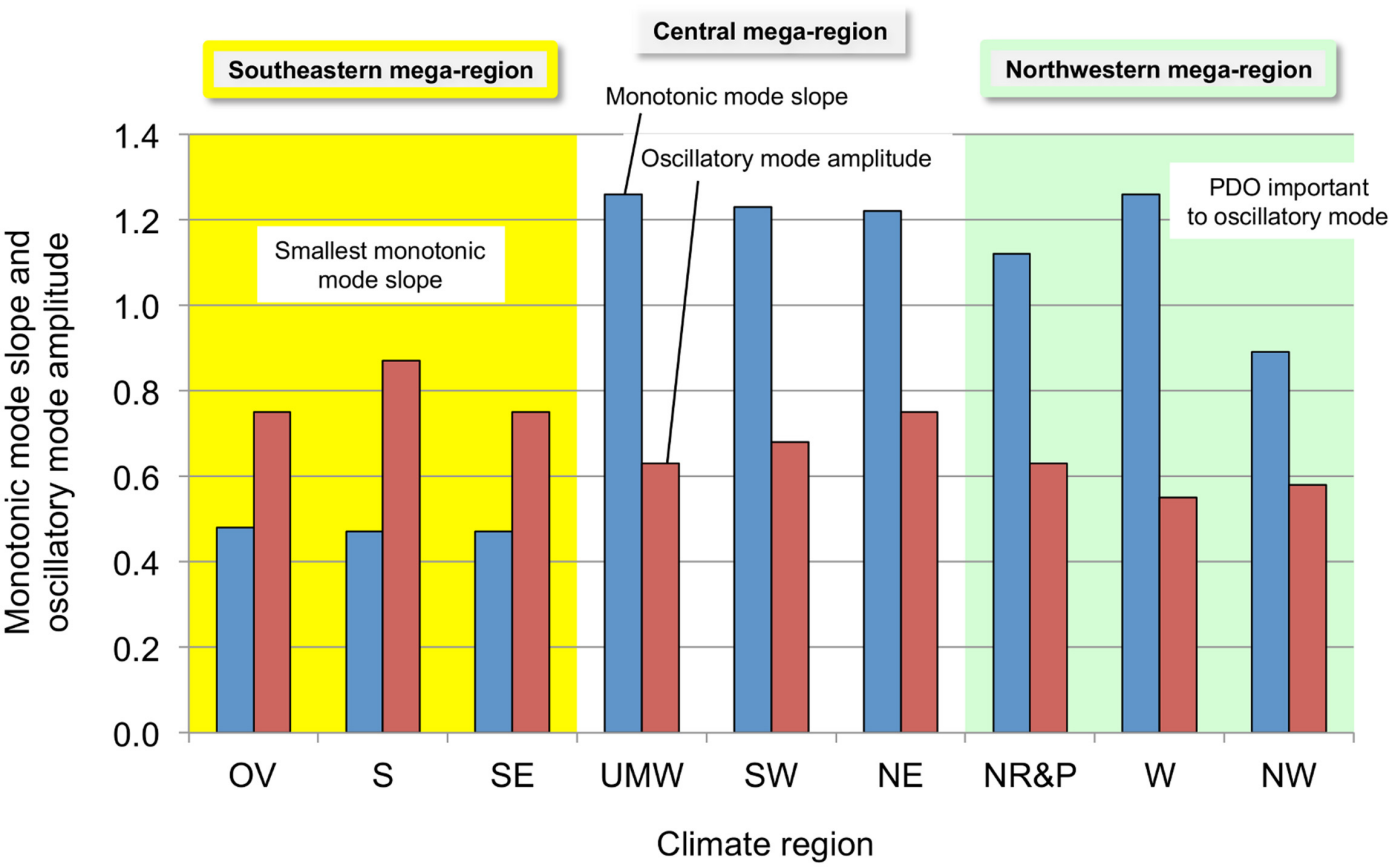
Monotonic mode slopes and oscillatory mode amplitudes. The slope of the monotonic mode (K yr^-1^×10^2^) is taken from the trend line of each temperature time series and the oscillatory mode peak-to-peak amplitude (K) is that required to maximize the correlation of the temperature time series with the calculated curve of temperature vs. time. The climate mega-regions are shown by the colors.

Comparing the slopes of the monotonic mode and the amplitude of the adjusted oscillatory mode reveals interesting information, shown by Fig 6. The nine NCDC climate regions fall into three groups: OV, S, and SE—where the monotonic mode slopes are much smaller than for the other climate regions; NR&P, W, and NW—where the PDO is a significant part of the oscillatory mode; and UMW, SW, and NE—where neither of these circumstances apply. The oscillatory mode amplitude decreases from 0.79 K for the OV, S, and SE group to 0.68 K for the UMW, SW, and NE group to 0.59 K for the NR&P, W, and NW group. These grouped climate regions constitute climate “mega-regions” differentiated by the degree to which they are influenced by the monotonic mode, the AMO oscillatory mode, and the PDO oscillatory mode. The names chosen for these mega-regions are: *Southeastern* (yellow background), *Central* (white background), and *Northwestern* (green background). The *Southeastern* and *Northwestern* mega-regions occupy the southeastern and northwestern corners of the contiguous U.S., while the *Central* mega-region occupies the space between these two sections, stretching from the southwest to the northeast.

**Fig 7:**
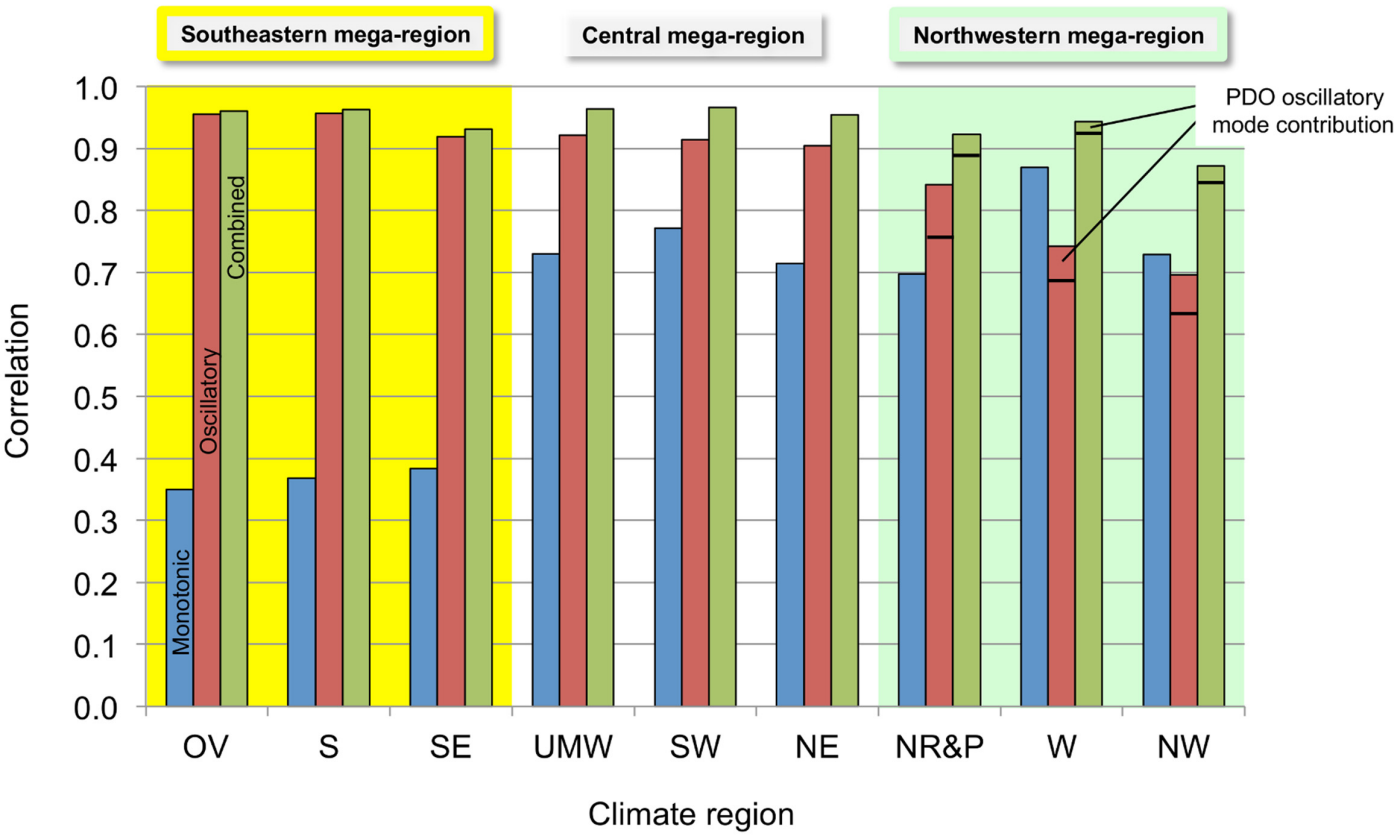
Correlations of the regional temperature time series with monotonic, oscillatory, and combined modes. This bar chart shows the correlations of temperature time series for individual climate regions with the monotonic, oscillatory, and combined modes. The climate mega-regions are shown by the colors.

Fig 7 shows the correlations of the smoothed time series with the calculated monotonic, oscillatory, and combined modes for each of the contiguous U.S. climate regions. In the *Southeastern* mega-region the correlation of the regional temperature time series with the monotonic mode is much smaller relative to the correlation with the oscillatory mode than is the case for the other climate regions. Correlation with the PDO is not significant (≤ 0.10) in either the *Southeastern* or *Central* mega-regions. In the *Northwestern* mega-region the time series is significantly correlated with the PDO: 0.32, 0.24, and 0.26 (not shown by the figure) for the NR&P, W, and NW climate regions, respectively. Adding a PDO contribution to the oscillatory mode, with a weight equal to about 50% of the AMO contribution, improves the correlation of the temperature time series, as shown by the lines near the tops of the *Northwestern* mega-region bars on Fig 7. The NR&P climate region is placed in the *Northwestern* mega-region on this basis, but it could also fit in the *Central* mega-region since its correlation with the oscillatory mode is higher than with the monotonic mode, in contrast to the W and NW climate regions. This suggests that the NR&P climate region should be divided into two parts, the western part assigned to the *Northwestern* mega-region, the eastern part to the *Central* mega-region.

**Fig 8:**
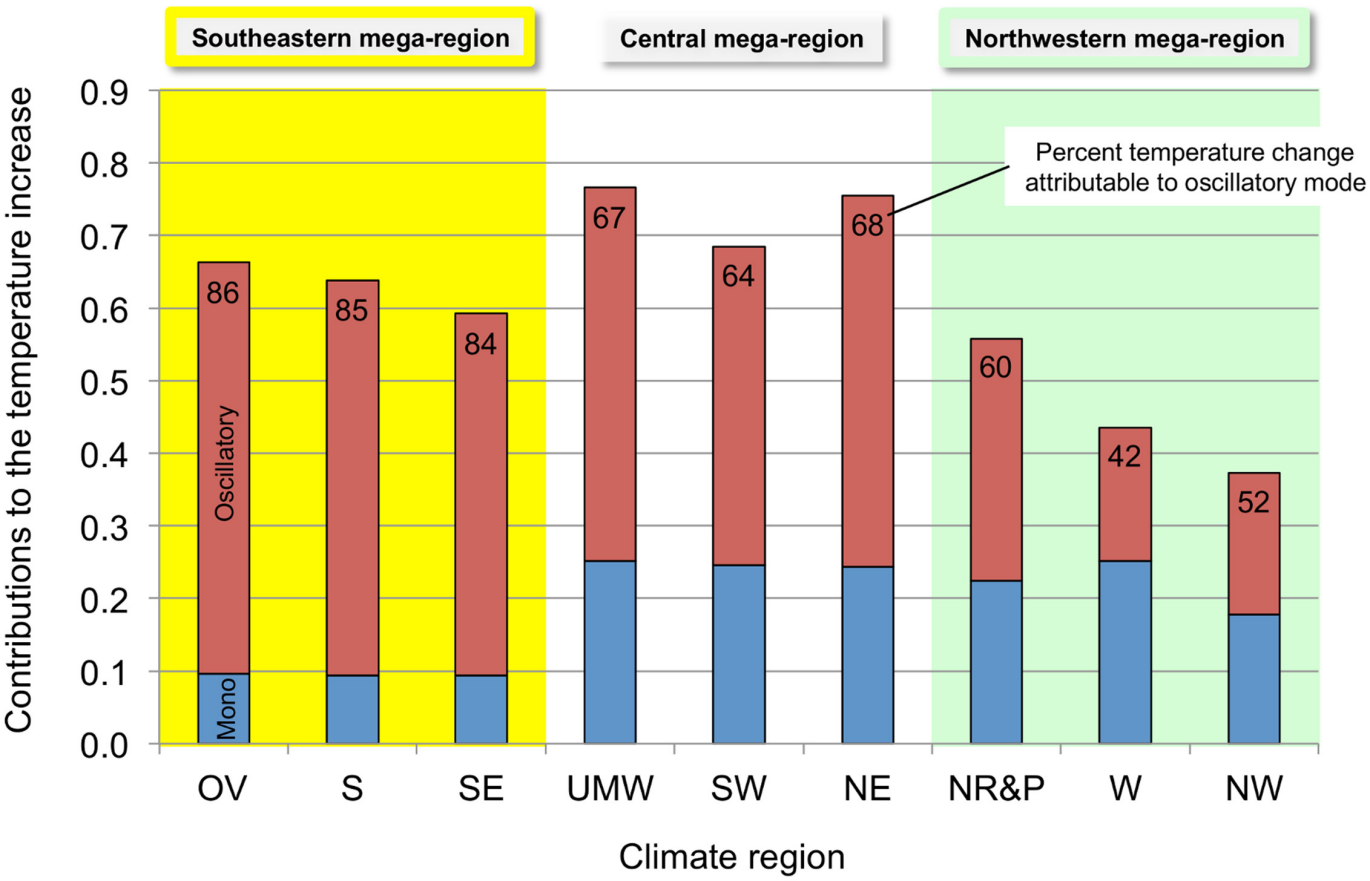
Contributions of the monotonic and oscillatory modes to the 1980–2000 contiguous U.S. regional temperature increases. The stacked bars show the contributions (K) of the monotonic and oscillatory modes for each climate region. The inset numbers are the percent of the total temperature increase attributable to the oscillatory mode. The climate mega-regions are shown by the colors.

Fig 8 shows the contribution of the calculated monotonic and oscillatory modes to the 1980–2000 increase in atmospheric temperature for the individual climate regions. The oscillatory mode is responsible for about 85% of the temperature increase in the *Southeastern* mega-region, compared to about 67% in the *Central* mega-region. The *Southeastern* mega-region has the smallest absolute monotonic mode contribution. Again, the NR&P doesn’t fit exactly into either the W or NW climate region since the oscillatory mode in the NR&P accounts for 60% of the temperature increase compared to 42 and 52% in the W and NW, respectively.

**Fig 9:**
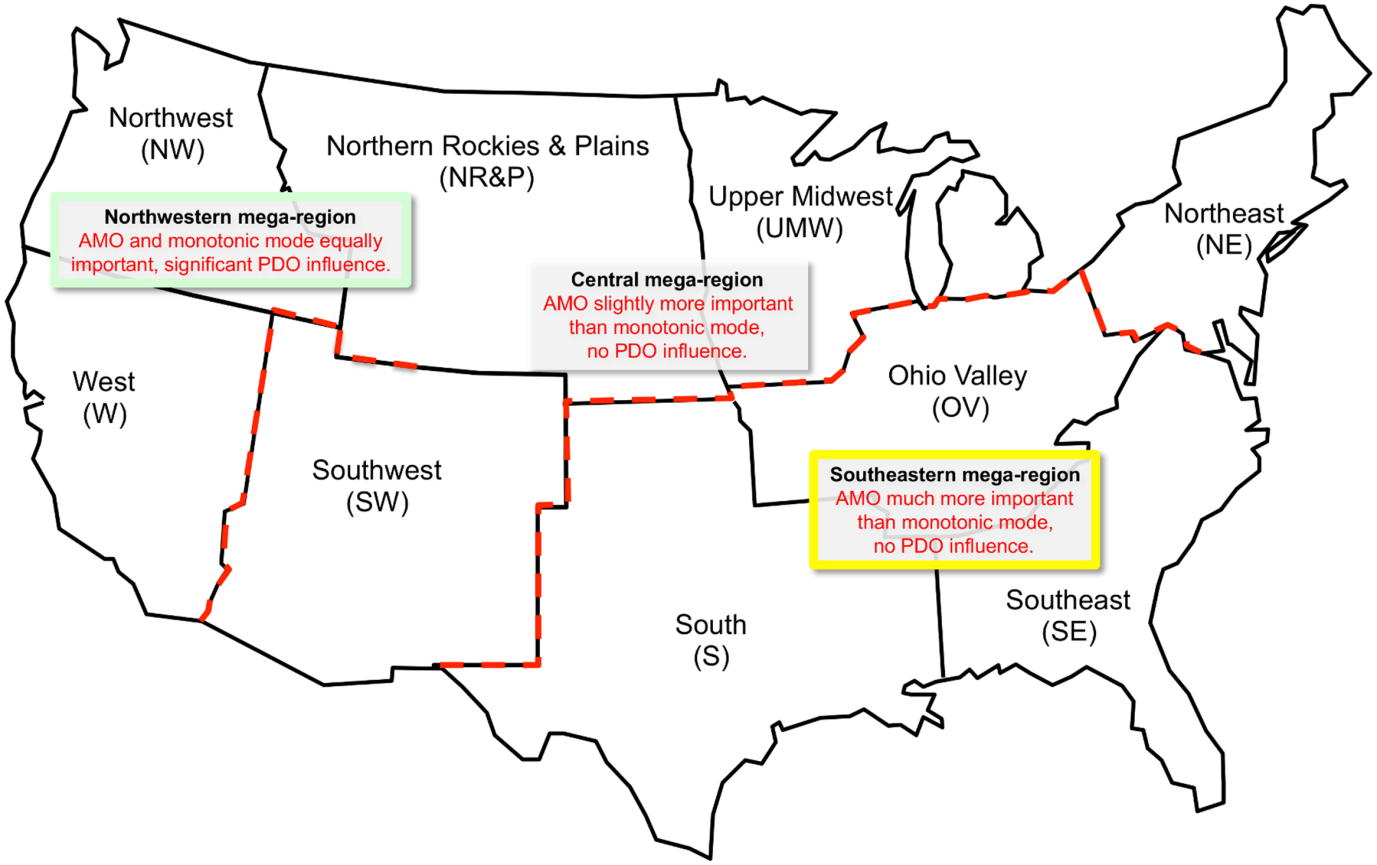
Contiguous U.S. climate mega-regions. The three mega-regions are shown relative to the approximate locations of the NCDC contiguous U.S. climate regions. Within the NR&P climate region the location of the boundary separating the *Northwestern* and *Central* mega-regions is unclear and is, accordingly, left undrawn.

Fig 9 shows the mega-region boundaries (red dashed lines) corresponding to the approximate locations of the NCDC climate regions comprising the mega-regions. The NR&P climate region should probably be divided between the *Central* and *Northwestern* mega-regions, but the location of the boundary separating those mega-regions within the NR&P is unknown. The figure also summarizes the mega-region characteristics in terms of oscillatory and monotonic mode influence.

## Conclusions

The 1935–2006 atmospheric temperature time series for the contiguous U.S. as a whole, and for each of the NCDC climate regions therein, are reproduced very well by the superposition of oscillatory and monotonic modes of temperature variation. The oscillatory modes represent primarily the AMO but also include a contribution from the PDO in some climate regions. The monotonic mode is presumed to be mostly the result of radiant forcing caused by increasing atmospheric CO_2_. The relative contributions of monotonic and oscillatory modes to the change in temperature are used to group the NCDC climate regions into three mega-regions. The W, NW, and NR&P climate regions, which together constitute the *Northwestern* mega-region, are characterized by a significant PDO contribution to the oscillatory mode. The PDO has a weight equal to about ½ that of the AMO in the combined oscillatory mode. The S, SE, and OV climate regions, which together constitute the *Southeastern* mega-region, are characterized by a monotonic mode slope (0.0047 K yr^-1^) much smaller than for the contiguous U.S. as a whole (0.0089 K yr^-1^). This implies that the AMO (SST) influence is so strong that it suppresses the rate of monotonic mode atmospheric temperature increase through the slow rate of increase in North Atlantic SST (0.0026 K yr^-1^). The SW, UMW, and NE, which constitute the *Central* mega-region, have neither of these characteristics.

Temperature time series correlations with the combined monotonic and oscillatory modes are 0.93–0.96 within the *Southeastern* mega-region and 0.95–0.97 within the *Central* mega-region. Within the *Northwestern* mega-region they are 0.92 and 0.94 for the NR&P and W, but only 0.87 for the NW. These correlations are reduced, to 0.87, 0.92, and 0.84, respectively, by removing the PDO from the oscillatory modes. This suggests that the NW climate region is subject to important influences other than those identified here.

The 1938–1974 decreases in contiguous U.S. temperatures are consistent with the superposition of the *downward*-trending oscillatory mode (dominated by the AMO) on the *upward*-trending monotonic mode. The contribution of the monotonic mode is smallest and that of the oscillatory mode is largest in the *Southeastern* mega-region. Similarly, the 1980–2000 increases in temperature are consistent with the superposition of the *upward*-trending oscillatory modes on the *upward*-trending monotonic mode. For the *Southeastern* mega-region the temperature increase is 0.59–0.66 K, about 85% of which is attributable to the AMO oscillatory mode. For the *Central* mega-region the temperature increase is 0.68–0.76 K, about 66% of which is attributable to the oscillatory mode. For the *Northwestern* mega-region the temperature increase is 0.37–0.56 K, 42–60% of which is attributable to the oscillatory mode. For the entire contiguous U.S. the temperature increase is 0.64 K, 72% of which is attributable to the AMO oscillatory mode.

The conclusion is that the oscillatory mode (mostly due to the AMO) is significantly more important than the monotonic mode (mostly due to increasing atmospheric CO_2_) in explaining the 1980–2000 temperature increase. The influence of the AMO is smaller in climate regions further removed from its vicinity. As to the pause in warming since 2000, it seems likely that this is the result of a recurrence of the AMO stage that initiated the 1938–1974 pause, the transition from a warming to a cooling AMO.

## Supporting Information

S1 File Principal component analysis for contiguous U.S. regional temperatures. (PPTX)
