# Supplementary material for: The Effect of Natural Multidecadal Ocean Temperature Oscillations on Contiguous U.S. Regional Temperatures
Source: PLoS One. 2015 Jun 22;10(6):e0131349. doi: 10.1371/journal.pone.0131349 (PMC4476671; doi:10.1371/journal.pone.0131349)
Supplement: S1 File — (PPTX) [file pone.0131349.s001.pptx]

## Slide 1
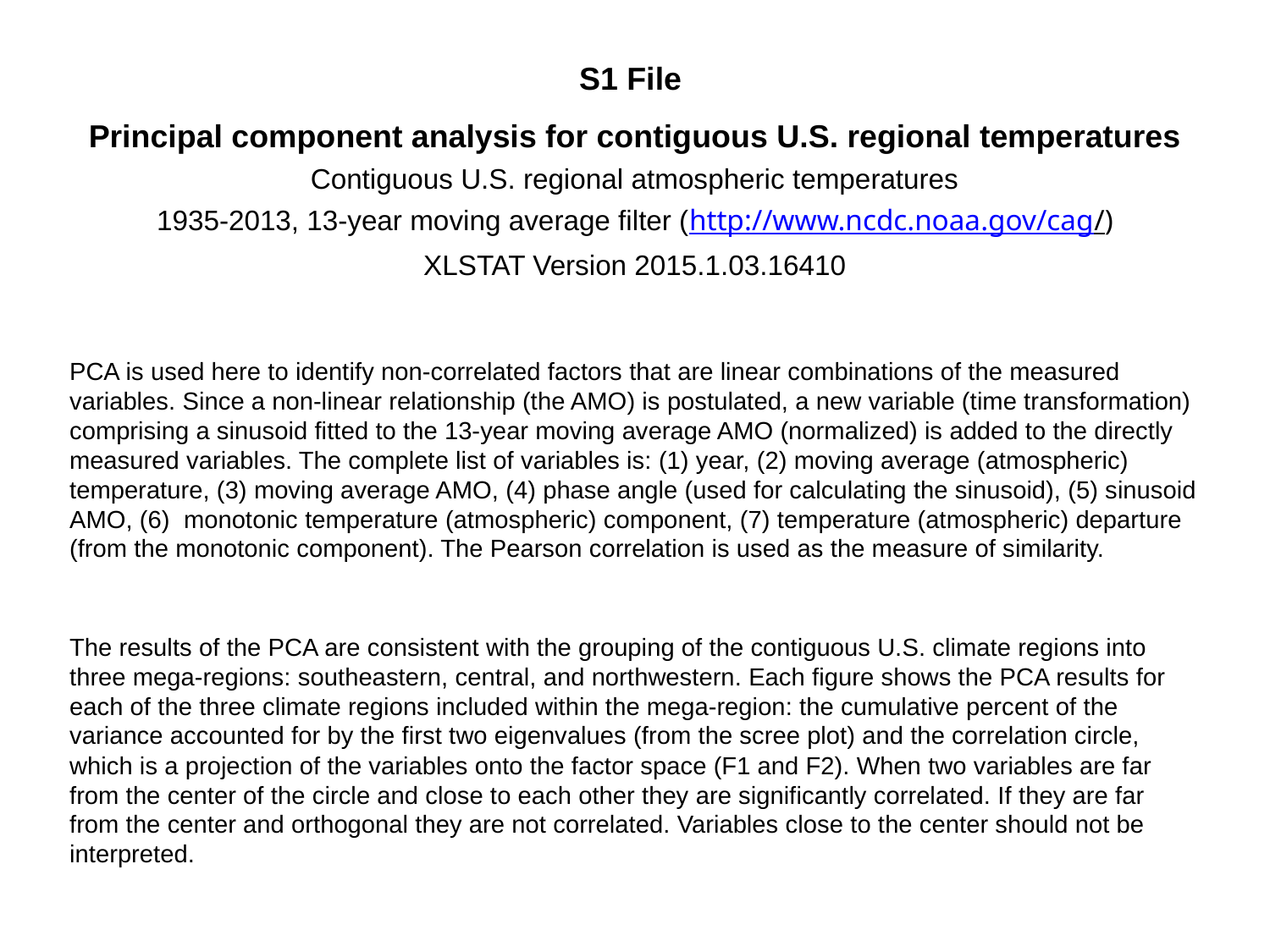

S1 File
Principal component analysis for contiguous U.S. regional temperatures
Contiguous U.S. regional atmospheric temperatures
1935-2013, 13-year moving average filter (http://www.ncdc.noaa.gov/cag/)
XLSTAT Version 2015.1.03.16410
PCA is used here to identify non-correlated factors that are linear combinations of the measured variables. Since a non-linear relationship (the AMO) is postulated, a new variable (time transformation) comprising a sinusoid fitted to the 13-year moving average AMO (normalized) is added to the directly measured variables. The complete list of variables is: (1) year, (2) moving average (atmospheric) temperature, (3) moving average AMO, (4) phase angle (used for calculating the sinusoid), (5) sinusoid AMO, (6) monotonic temperature (atmospheric) component, (7) temperature (atmospheric) departure (from the monotonic component). The Pearson correlation is used as the measure of similarity.
The results of the PCA are consistent with the grouping of the contiguous U.S. climate regions into three mega-regions: southeastern, central, and northwestern. Each figure shows the PCA results for each of the three climate regions included within the mega-region: the cumulative percent of the variance accounted for by the first two eigenvalues (from the scree plot) and the correlation circle, which is a projection of the variables onto the factor space (F1 and F2). When two variables are far from the center of the circle and close to each other they are significantly correlated. If they are far from the center and orthogonal they are not correlated. Variables close to the center should not be interpreted.

## Slide 2
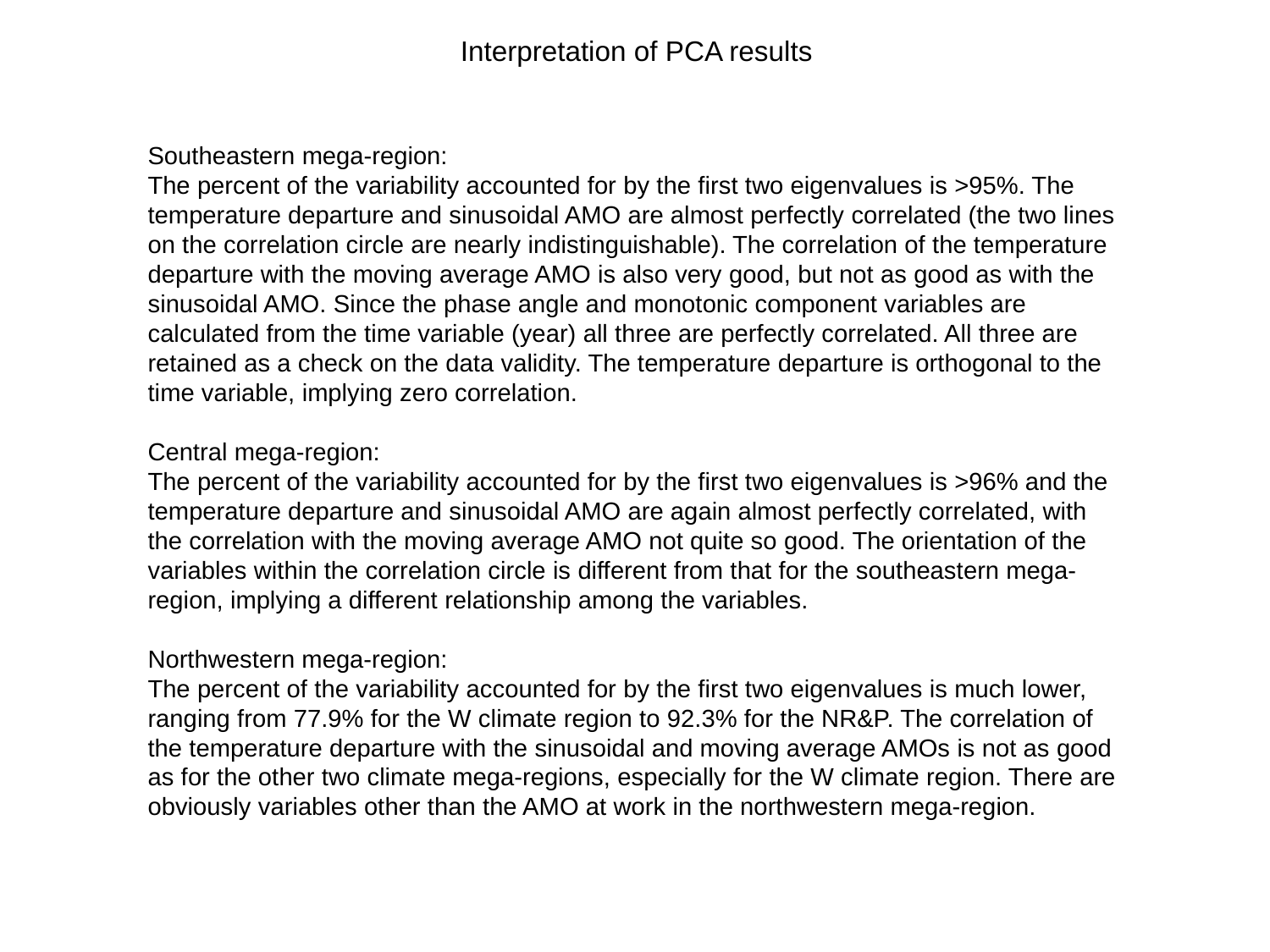

Interpretation of PCA results
Southeastern mega-region:
The percent of the variability accounted for by the first two eigenvalues is >95%. The temperature departure and sinusoidal AMO are almost perfectly correlated (the two lines on the correlation circle are nearly indistinguishable). The correlation of the temperature departure with the moving average AMO is also very good, but not as good as with the sinusoidal AMO. Since the phase angle and monotonic component variables are calculated from the time variable (year) all three are perfectly correlated. All three are retained as a check on the data validity. The temperature departure is orthogonal to the time variable, implying zero correlation.
Central mega-region:
The percent of the variability accounted for by the first two eigenvalues is >96% and the temperature departure and sinusoidal AMO are again almost perfectly correlated, with the correlation with the moving average AMO not quite so good. The orientation of the variables within the correlation circle is different from that for the southeastern mega-region, implying a different relationship among the variables.
Northwestern mega-region:
The percent of the variability accounted for by the first two eigenvalues is much lower, ranging from 77.9% for the W climate region to 92.3% for the NR&P. The correlation of the temperature departure with the sinusoidal and moving average AMOs is not as good as for the other two climate mega-regions, especially for the W climate region. There are obviously variables other than the AMO at work in the northwestern mega-region.

## Slide 3
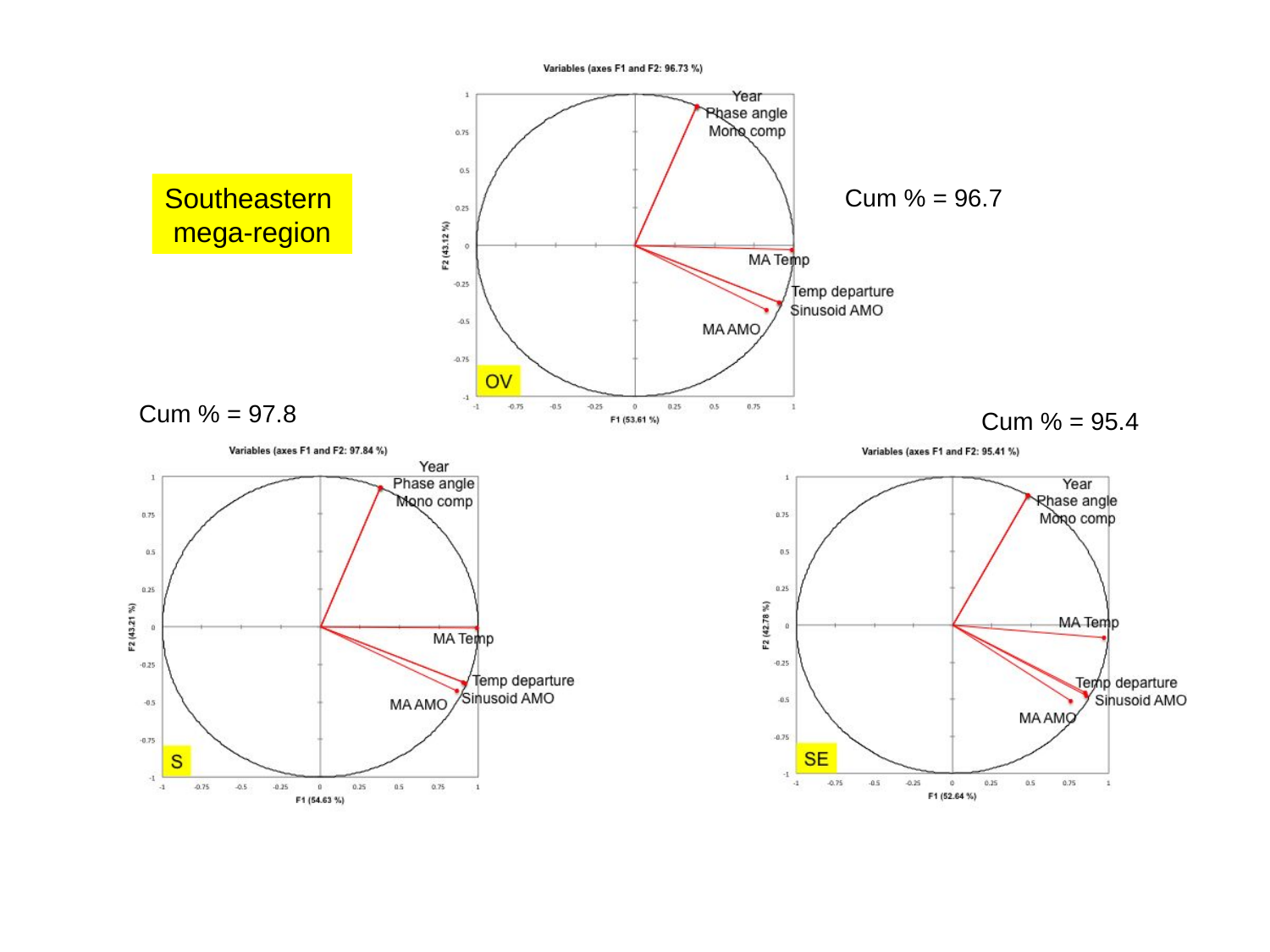

Southeastern
mega-region
Cum % = 96.7
Cum % = 97.8
Cum % = 95.4

## Slide 4
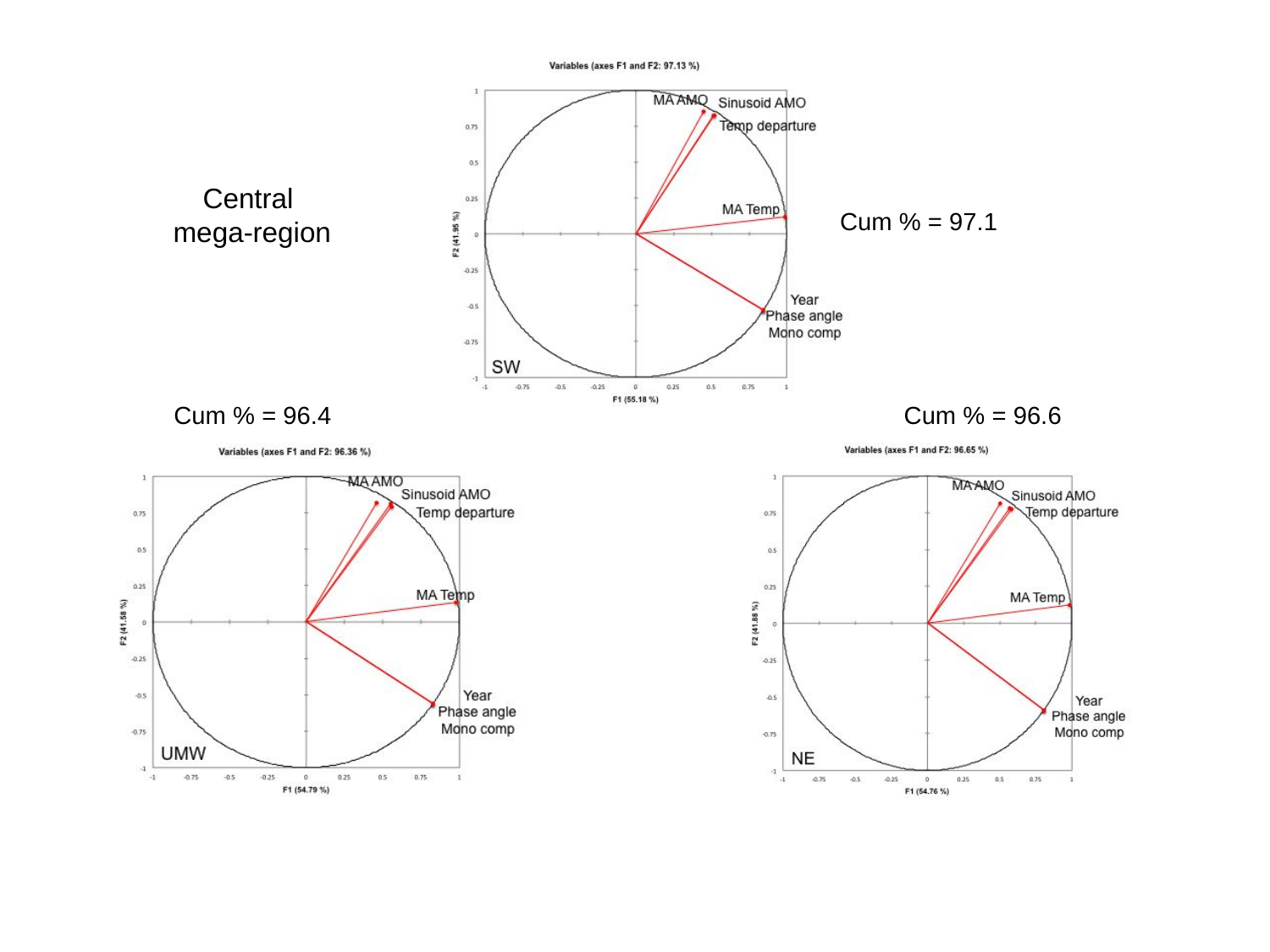

Central
mega-region
Cum % = 97.1
Cum % = 96.4
Cum % = 96.6

## Slide 5
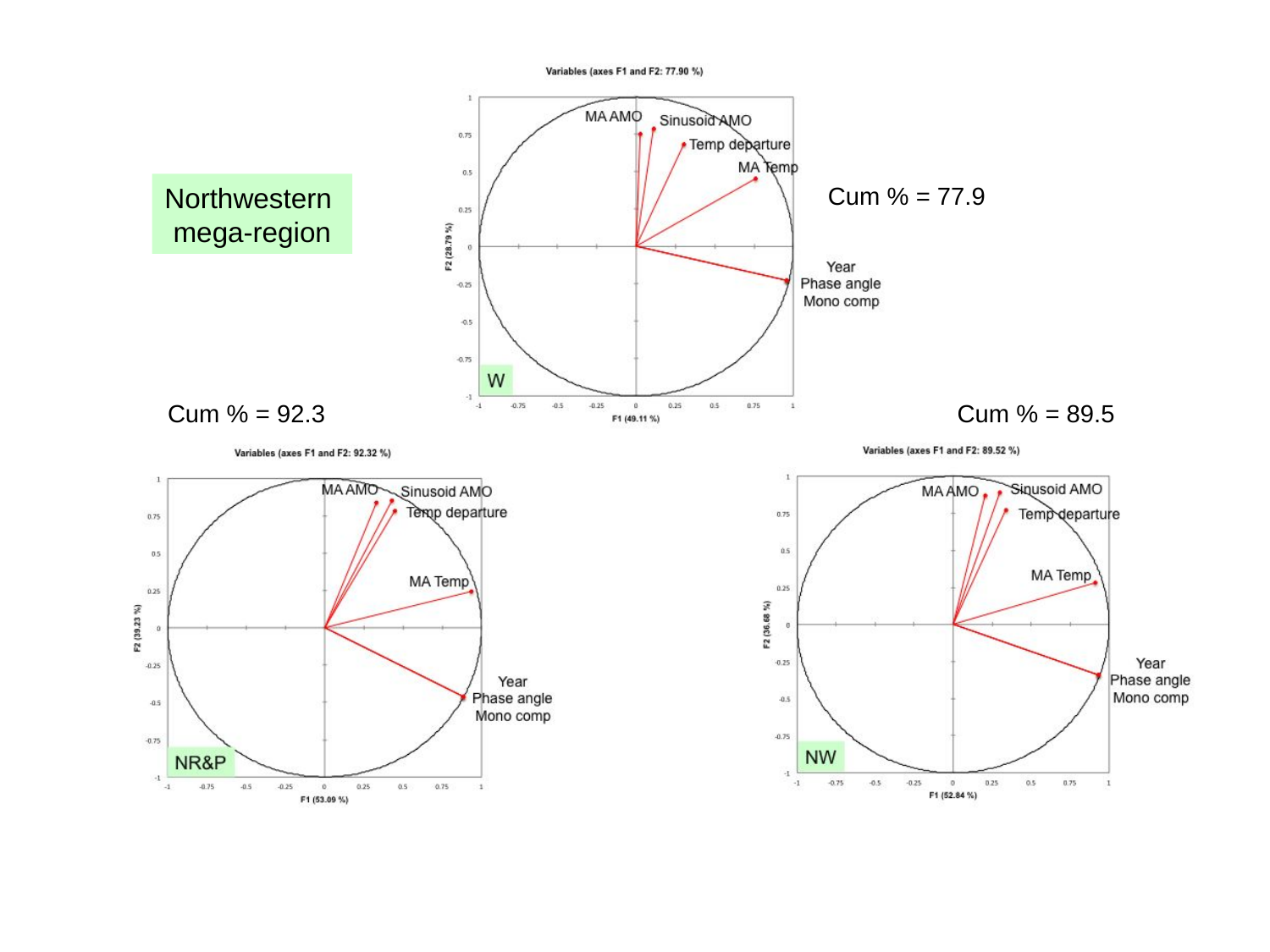

Northwestern
mega-region
Cum % = 77.9
Cum % = 92.3
Cum % = 89.5
